# Redox‐Polymer‐Wired [NiFeSe] Hydrogenase Variants with Enhanced O_2_ Stability for Triple‐Protected High‐Current‐Density H_2_‐Oxidation Bioanodes

**DOI:** 10.1002/cssc.202000999

**Published:** 2020-06-08

**Authors:** Adrian Ruff, Julian Szczesny, Maria Vega, Sonia Zacarias, Pedro M. Matias, Sébastien Gounel, Nicolas Mano, Inês A. C. Pereira, Wolfgang Schuhmann

**Affiliations:** ^1^ Analytical Chemistry—Center for Electrochemical Sciences (CES) Faculty of Chemistry and Biochemistry Ruhr-University Bochum Universitätsstr. 150 44780 Bochum Germany; ^2^ Facultat de Biociències Universitat Autònoma de Barcelona (UAB) Carrer de la Vall Moronta 08193 Bellaterra Spain; ^3^ Instituto de Tecnologia Química e Biológica António Xavier (ITQB NOVA) Universidade NOVA de Lisboa Av. da República 2780-157 Oeiras Portugal; ^4^ Instituto de Biologia Experimental e Tecnológica (iBET) Apartado 12 2780-901 Oeiras Portugal; ^5^ CNRS CRPP, UMR 5031 Univ. Bordeaux 33600 Pessac France

**Keywords:** bioelectrocatalysis, biofuel cells, enzyme engineering, hydrogenases, redox polymers

## Abstract

Variants of the highly active [NiFeSe] hydrogenase from *D. vulgaris* Hildenborough that exhibit enhanced O_2_ tolerance were used as H_2_‐oxidation catalysts in H_2_/O_2_ biofuel cells. Two [NiFeSe] variants were electrically wired by means of low‐potential viologen‐modified redox polymers and evaluated with respect to H_2_‐oxidation and stability against O_2_ in the immobilized state. The two variants showed maximum current densities of (450±84) μA cm^−2^ for G491A and (476±172) μA cm^−2^ for variant G941S on glassy carbon electrodes and a higher O_2_ tolerance than the wild type. In addition, the polymer protected the enzyme from O_2_ damage and high‐potential inactivation, establishing a triple protection for the bioanode. The use of gas‐diffusion bioanodes provided current densities for H_2_‐oxidation of up to 6.3 mA cm^−2^. Combination of the gas‐diffusion bioanode with a bilirubin oxidase‐based gas‐diffusion O_2_‐reducing biocathode in a membrane‐free biofuel cell under anode‐limiting conditions showed unprecedented benchmark power densities of 4.4 mW cm^−2^ at 0.7 V and an open‐circuit voltage of 1.14 V even at moderate catalyst loadings, outperforming the previously reported system obtained with the [NiFeSe] wild type and the [NiFe] hydrogenase from *D. vulgaris* Miyazaki F.

## Graphical abstract

**Triple protection**: A stable, high‐current‐density‐based H_2_‐oxidation bioanode is presented. It is equipped with [NiFeSe] variants that show enhanced O_2_ tolerance, which are immobilized and wired to electrode surfaces with a low‐potential viologen‐modified polymer. The polymer acts simultaneously as a high‐potential and O_2_ shield. The triply protected bioanodes are incorporated in membrane‐free biofuel cells, which reveal benchmark performances at moderate catalyst loading.

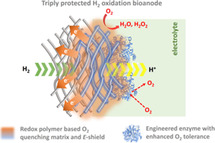

## Introduction

Bioelectrocatalysis has gained huge importance in the past decades and is considered a promising research field for the development of novel sustainable energy conversion and storage systems^1^ as well as for the green production of value‐added chemicals and (solar) fuels.^1^, ^2^ In particular, the use of biocatalysts to produce sustainable H_2_/O_2_‐powered biofuel cells with high power output has become a major research area in this context. Although the biocathodes used for such devices are mainly based on rather robust, stable, and easy‐to‐wire multicopper oxidases such as bilirubin oxidase or laccase,^3^ the biocatalyst at the bioanode, that is, highly active H_2_‐oxidizing Ni/Fe‐based hydrogenases, typically suffers from pronounced O_2_ sensitivity and fast inactivation at high potentials especially under anode‐limiting conditions.^4^ Hence, the preparation of hydrogenase‐based bioanodes requires the implementation of specific protection systems, for example, based on O_2_‐reducing low‐potential redox polymers, or the use of O_2_‐tolerant but usually less active hydrogenases, such as, for instance, the hydrogenases from *Escherichia coli*,^5^ *Ralstonia eutropha*,^6^ or *Aquifex aeolicus*.^7^ The introduction of O_2_‐reducing redox polymers for electrical wiring and protection of air‐sensitive hydrogenases was successfully demonstrated for various hydrogenases including [NiFe] hydrogenase from *D. vulgaris* Miyazaki F,^8^ [FeFe] hydrogenase from *Chlamydomonas reinhardtii*,^9^ and [NiFeSe] hydrogenase from *D. vulgaris* Hildenborough.^10^ Moreover, effective protection was even observed for thin films^11^ and polymer/enzyme‐modified gas‐diffusion electrodes (GDEs), which could be incorporated in membrane‐free biofuel cells that exhibited benchmark power densities for polymer‐based systems.^12^ In addition, the low‐potential redox polymer not only acts as an O_2_‐quenching matrix but also as a Nernst buffer system for the biocatalyst, preventing inactivation at high potentials.^8^ Hence, low‐potential redox polymers provide a double protection system for such sensitive materials, which was even transposable to synthetic catalysts.^13^ Moreover, hydrogenases deactivated under aerobic conditions may be reactivated by the low‐potential polymer matrix, as was shown previously for [NiFe]^14^ and [NiFeSe]^10^ hydrogenases.

Although O_2_‐tolerant hydrogenases derived from the above‐mentioned microorganisms can be operated in the presence of distinct levels of O_2_,^15^ inactivation at high potentials still remains an issue.^5^, ^6^, ^7^ Furthermore, the overpotential for H_2_‐oxidation is often at more positive values than those of O_2_‐sensitive [NiFe] and [FeFe] hydrogenases.^5^, ^15^, ^16^ Evidently, this will limit the power output of a related biofuel cell because this value will directly affect the maximum open‐circuit voltage (OCV). Hence, a combination of redox polymers (double protection shield) and hydrogenase variants with enhanced O_2_ tolerance would allow the development of *triple‐protected* bioanodes. We want to emphasize that although the redox polymer requires a slightly more positive redox potential than the hydrogenase itself to ensure successful electron exchange at oxidative conditions, the open‐circuit potential of the corresponding bioanode and, consequently, the OCV of the cell will not be limited by the mid‐point potential of the redox polymer owing to the pseudo‐capacitive properties of the polymer matrix.^17^ This effect was shown for glucose oxidase/bilirubin oxidase^18^ as well as hydrogenase/bilirubin oxidase‐based biofuel cells,^12^ in which the anodic catalyst (glucose oxidase, hydrogenase) was electrically wired by means of a redox polymer.

The properties of enzymes can be modulated by enzyme engineering.^19^ For instance, glucose oxidase that uses O_2_ as a natural electron acceptor could be turned into an O_2_‐insensitive enzyme by site‐directed mutagenesis^20^ and cofactor redesign.^21^ Artificial maturation of [FeFe] hydrogenase allows for the fine tuning of the properties of the active center of the enzyme.^22^ The synthetic nature of the active site allows for the incorporation of specific ligands or the alteration of the overall ligand sphere to adjust the properties of the whole enzyme.^23^, ^24^ Following this approach, the stability of [FeFe] hydrogenases could be enhanced, and the overall activity of the enzyme can be controlled.^23^, ^24^ Although the maturation process occurs spontaneously without any helper proteins or additional cofactors,^25^ the preparation of the active site of a [FeFe] hydrogenase requires distinct synthetic efforts,^26^ and the maturation process is an additional step in the preparation of the active enzyme. In contrast, once developed, variants of an enzyme showing altered properties are produced directly from the living organisms. The additional maturation step and complex synthesis is thus not required.

**Figure 1:**
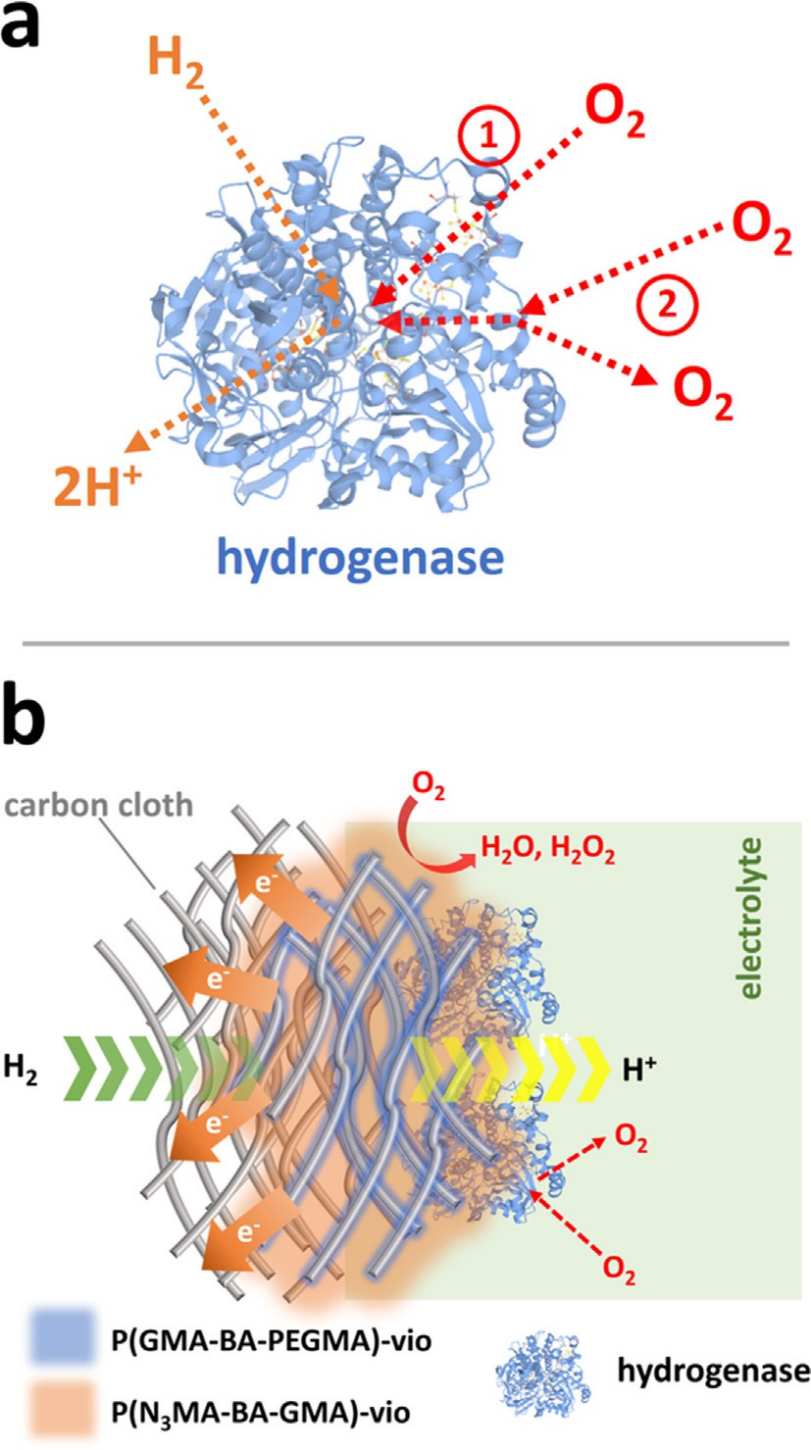
(a) Cartoon showing the mechanism of the different behavior of the wild type (**1**) and the variants (**2**) of [NiFeSe] hydrogenase when exposed to O_2_. In the wild‐type [NiFeSe] hydrogenase, oxygen reaches the active center of the enzyme (**1**), whereas in the variants G491A and G491S, the pathway to the active center is partially blocked by altered amino acid residues, which hamper the access of O_2_ (**2**). For a detailed structural and mechanistic description, see Ref. 27. (b) Schematic of the high‐current‐density carbon cloth gas‐diffusion H_2_‐oxidation bioanode equipped with a polymer/hydrogenase layer. For the immobilization of the active P(N_3_MA‐BA‐GMA)‐vio/hydrogenase layer, a carbon cloth‐based gas‐diffusion layer was first modified with an adhesion layer, that is, the redox polymer P(GMA‐BA‐PEGMA)‐vio, which shows a higher hydrophobic monomer content (for a detailed description of the electrode architecture and electrochemical gas diffusion cell, see Ref. 12). By combining the low‐potential redox polymer with a more O_2_‐tolerant hydrogenase variant, a stable high‐current‐density bioanode is obtained, which can be operated in a membrane‐free H_2_/O_2_ biofuel cell. (a, b) The structure of the *wt*‐[NiFeSe] from *D. vulgaris* Hildenborough (5JSH)^28^ was used as a representative enzyme model; not drawn to scale.

Recently, it was shown that variants (G491A and G491S) of the [NiFeSe] hydrogenase from *D. vulgaris* Hildenborough with modification in a specific amino acid close to the active site led to enhanced stability of the biocatalyst in the presence of O_2_ while retaining a high activity for H_2_‐oxidation [G491A: up to (4080±80) s^−1^; G491S: up to (2810±150) s^−1^, wild type: ≈(4850±260) s^−1^] and a redox potential that is still close to the H_2_/2 H^+^ couple [approximately −450 mV vs. standard hydrogen electrode (SHE) at pH 7].^27^ The enhanced stability of the altered proteins was attributed to a physical blocking effect of the O_2_ molecule in a hydrophilic channel that connects the active site of the protein with the enzyme surface, thus preventing oxidation of a specific active‐site cysteine ligand (Figure 1 a; for a more comprehensive description of the structural changes inside the protein shell of the enzyme that lead to the desired O_2_‐blocking effect, see Ref. 27). This effect was evidenced by protein film electrochemistry conducted in a direct electron transfer (DET) regime and in the presence and absence of O_2_.^27^ However, the DET mode does not provide any protection against high‐potential inactivation or against high O_2_ concentrations and is hence impractical for potential applications. Nevertheless, it shows that variants of this type of hydrogenase can be prepared with enhanced O_2_ stability.

In this contribution, we combined the advantages of the enhanced O_2_‐stable variants of [NiFeSe] hydrogenase from *D. vulgaris* Hildenborough and the O_2_ quenching and Nernst buffer properties of low‐potential viologen‐modified redox polymers to fabricate high‐current‐density bioanodes, which could be successfully incorporated into H_2_/O_2_‐powered biofuel cells exhibiting benchmark power densities at moderate catalyst loadings.

## Results and Discussion

### Electrical wiring

The redox polymer P(N_3_MA‐BA‐GMA)‐vio [poly(3‐azido‐propyl methacrylate‐*co*‐butyl acrylate‐*co*‐glycidyl methacrylate)‐vio, with vio=1‐(5‐hexyn‐1‐yl)‐1′‐methyl‐4,4′‐bipyridinium; see Figure S1 in the Supporting Information] was used previously for productive electrical wiring and protection of wild‐type [NiFeSe] hydrogenase from *D. vulgaris* Hildenborough (*wt*‐[NiFeSe]).^10^ Benchmark H_2_‐oxidation currents of approximately 1.7 mA cm^−2^ at optimized conditions were obtained for P(N_3_MA‐BA‐GMA)‐vio/*wt*‐[NiFeSe]‐modified glassy carbon electrodes.^10^ Stimulated by our previous findings, we exploited the possibility to electrically wire two O_2_‐tolerant [NiFeSe] variants, namely G491A and G491S, to the same electrode material.

**Figure 2:**
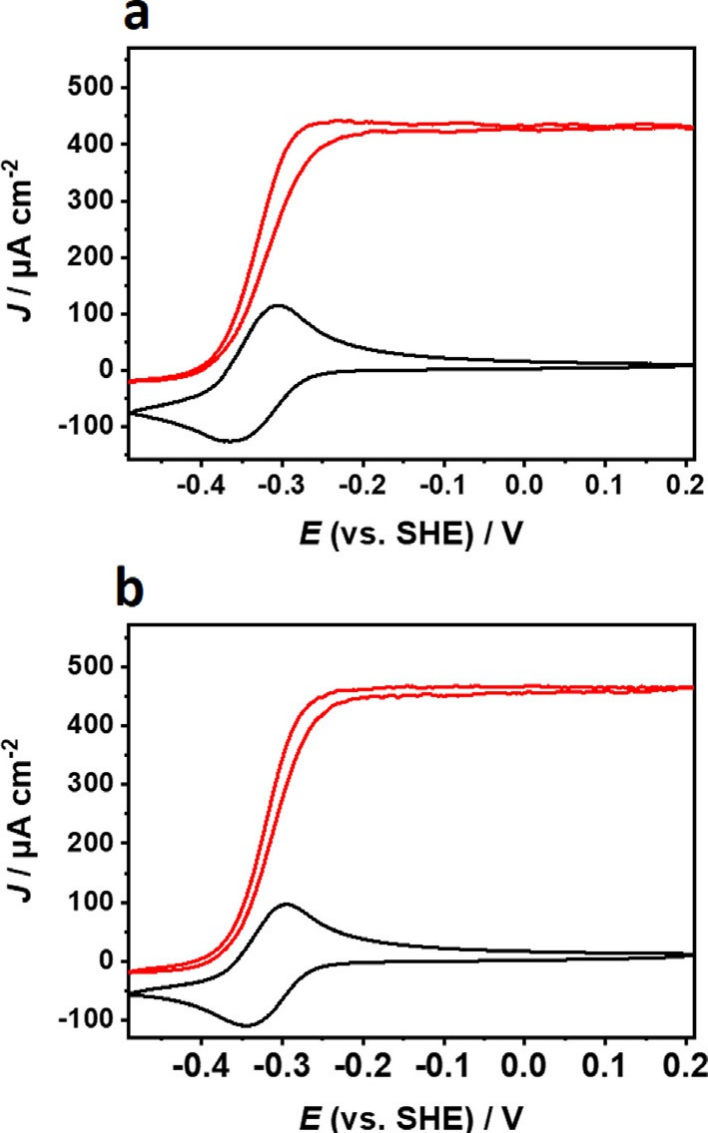
Cyclic voltammograms recorded at a scan rate of 10 mV s^−1^ of (a) P(N_3_MA‐BA‐GMA)‐vio/G491A and (b) P(N_3_MA‐BA‐GMA)‐vio/G491S films immobilized on glassy carbon disk electrodes (3 mm Ø) in the absence (black lines, 100 % Ar, purged through solution) and presence of H_2_ (red lines, 100 % H_2_, purged through solution). Working electrolyte: phosphate buffer, 0.1 m, pH 7.3, room temperature.

Indeed, cyclic voltammograms of drop‐cast P(N_3_MA‐BA‐GMA)‐vio/G419A and P(N_3_MA‐BA‐GMA)‐vio/G419S films measured under turnover conditions, that is, under H_2_ atmosphere (Figure 2 a, b, red curves), showed pronounced catalytic H_2_‐oxidation waves with half‐wave potentials (a and b: approximately −0.32 V vs. SHE), which matches closely the mid‐point potential of the polymer‐bound viologen units (≈0.34 V vs. SHE, black curves). The behavior is in line with the results measured for the wild type (Figure S2 in the Supporting Information). We hence conclude that the [NiFeSe] variants can also be productively wired through the redox polymer P(N_3_MA‐BA‐GMA)‐vio in a mediated electron‐transfer regime. Moreover, long‐term chronoamperometric measurements over 7 h under continuous turnover conditions showed similar operational stability for the wild type and the two variants (Figure S3 in the Supporting Information).

The maximum current densities *J*_max_ measured for the individual, freshly prepared electrodes were calculated as average values from three electrodes as (450±84) μA cm^−2^ for G491A and (476±172) μA cm^−2^ for G941S. The wild type shows a *J*_max_ value of (752±259) μA cm^−2^ (note that for the wild type and the variants, storage of the modified electrodes leads to a decrease in overall electrode activity). The results are in line with the measured activities of the used enzyme batches [G491A: (1918±119) s^−1^ and G491S: (2416±387) s^−1^; note that the standard deviations are overlapping], which are below the activity of the wild‐type enzyme (4850±260 s^−1^)^27^ and below the maximum values reported previously for the variants (see values above).^27^

The steady‐state current observed at high potentials (>−0.2 V vs. SHE, Figure 2, red lines) under turnover conditions indicates that the variants can also be effectively protected against high‐potential inactivation in contrast to the operation under DET conditions for this type of hydrogenases.^29^, ^30^

### Oxygen tolerance

Because the low‐potential redox polymer acts as an O_2_‐quenching matrix and thus protects the enzyme from O_2_‐damage,^8^ the stability of the variants and the wild type against oxygen in the immobilized sate was measured in the absence of H_2_ in chronoamperometric experiments. Under these conditions, electrons from H_2_‐oxidation are absent and cannot be used by the polymer matrix to reduce incoming O_2_.^8^, ^31^

**Figure 3:**
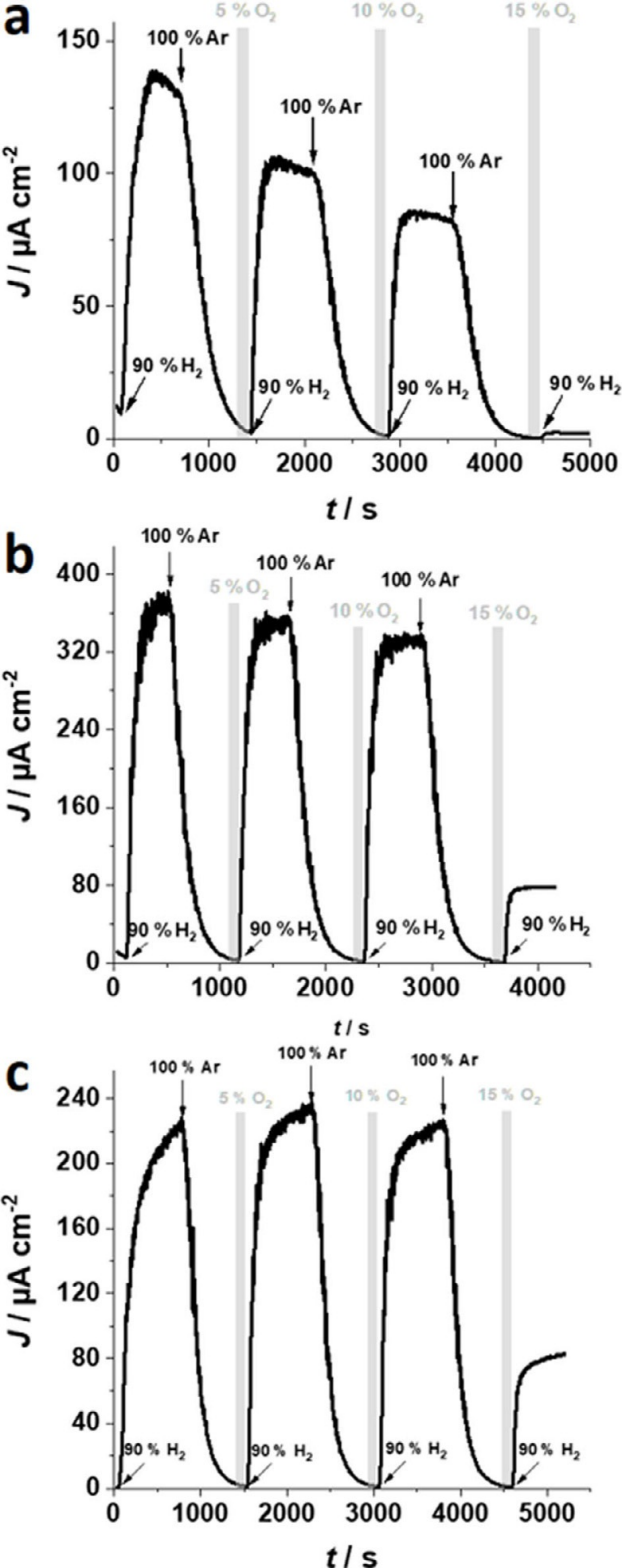
Representative chronoamperometric experiments with (a) P(N_3_MA‐BA‐GMA)‐vio/wt‐[NiFeSe], (b) P(N_3_MA‐BA‐GMA)‐vio/G491A, and (c) P(N_3_MA‐BA‐GMA)‐vio/G491S films immobilized on glassy carbon disk electrodes (3 mm Ø) under alternating gas feeds (90 % H_2_/10 % Ar, 100 % Ar as well as 5 % O_2_/95 %Ar, 10 % O_2_/90 % Ar, and 15 % O_2_/85 % Ar). Working conditions: phosphate buffer, 0.1 m, pH 7.3, room temperature; *E*_appl_=+160 mV vs. SHE.

Figure 3 shows chronoamperometric experiments at an applied potential (*E*_appl_) of +160 mV (vs. SHE) under alternating gas‐mixture atmospheres. The O_2_ content in the gas feed was stepwise increased after each H_2_ cycle. To ensure that all H_2_ had been removed before the O_2_ was added to the gas feed, the cell was purged with argon. After the background current was reached, the film was exposed to an O_2_/Ar mixture with varying O_2_ content (5, 10, and 15 %, gray shaded areas in Figure 3). The wild type shows a steady decrease of the H_2_‐oxidation activity over all O_2_/Ar cycles. After exposure to 15 % O_2_, the electrode remains inactive when switching the gas feed back to H_2_. This is consistent with a fast in‐diffusion of O_2_ to the active center of the enzyme (Figure 1 a). In contrast, both variants show a rather constant current output after the 5 and 10 % O_2_ cycle. Moreover, even after exposure to 15 % O_2_, both electrodes still show a remarkable activity towards H_2_‐oxidation (G491A: ≈20 % of the initial H_2_‐oxidation current; G491S: ≈35 %). The results demonstrate that the variants indeed exhibit an increased O_2_ tolerance compared with the wild‐type enzyme owing to a partial blocking of molecular oxygen (hampered access) based on the altered amino acid residues in the variants (Figure 1 a) and, by this, that the variants provide an additional protection for the proposed H_2_‐oxidation bioanodes. The electrochemical results obtained with the polymer/enzyme films are in line with the results reported for operating the same hydrogenases in the DET regime.^27^ However, strong variations in the residual currents were observed after exposure to O_2_, which is attributed to variations in film thickness and inhomogeneities of the catalytic layers, leading to different diffusion profiles of O_2_. However, in all experiments the variants showed a higher stability towards O_2_.

### Reactivation

For the wild‐type [NiFeSe] hydrogenase, which was deactivated under aerobic conditions, reactivation is known to occur quickly at rather negative potentials.^29^, ^30^ Moreover, the reduced low‐potential polymer matrix P(N_3_MA‐BA‐GMA)‐vio is also able to reactivate the inactive [NiFeSe] hydrogenase.^10^

**Figure 4:**
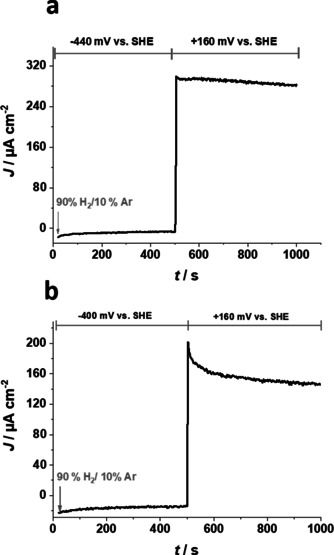
Chronoamperometric experiments with aerobically deactivated (a) P(N_3_MA‐BA‐GMA)‐vio/G491A and (b) P(N_3_MA‐BA‐GMA)‐vio/G491S films immobilized on glassy carbon disk electrodes (3 mm Ø). First, a potential of −440 mV vs. SHE was applied for 500 s to fully reduce the viologen‐modified polymer (the enzyme is reactivated during reduction via the polymer). After switching the potential to +160 mV vs. SHE (*t*>500 s), H_2_‐oxidation currents indicate successful reactivation. Working conditions: phosphate buffer, 0.1 m, pH 7.3, room temperature, electrodes were deactivated by extensive exposure to O_2_ until any H_2_‐oxidation current was absent.

To evaluate a possible reactivation behavior of the two [NiFeSe] hydrogenase variants, glassy carbon electrodes modified with P(N_3_MA‐BA‐GMA)‐vio/G491A and P(N_3_MA‐BA‐GMA)‐vio/G491S films were exposed to O_2_ until complete inactivation occurred, as evidenced by the current dropping back to background values. Application of a negative potential of −440 mV (vs. SHE; polymer is fully reduced, inactive mediator form) for 500 s leads to reactivation of the enzyme (Figure 4) as indicated by the oxidative currents, which were observed again when the potential was stepped back to +160 mV (vs. SHE; *t*>500 s, mediator is oxidized, active form). Both potentials were applied under a 90 % H_2_/10 % Ar gas feed. The wild type shows the same behavior (see Figure S4 in the Supporting Information and Ref. 10).

For practical applications, this effect is highly desirable because a potentially necessary exchange of a deactivated electrode in a device can be prevented. Instead, a short cathodic potential pulse might reactivate the electrode, and operation can be resumed.

### Membrane‐free H_2_/O_2_ biofuel cells

Oxygen‐tolerant hydrogenases typical display higher redox potentials, which will decrease the maximum OCV of a corresponding biofuel cell compared with their O_2_‐sensitive analogues. However, because the O_2_‐tolerant variants G491A and G941S show similar H_2_‐oxidation potentials as the wild type,^27^ the electrical wiring is possible with the same polymer (Figure 2). Hence, we expect similar OCV values for related biofuel cells as for those based on the wild‐type hydrogenase. To evaluate the performance of the bioanodes in a biofuel cell, polymer/hydrogenase‐modified glassy carbon electrodes were combined with a gas‐diffusion O_2_‐reducing bilirubin oxidase‐based biocathode. The use of a gas‐diffusion system at the cathode side, in which mass transport is not limiting, ensures bioanode‐limiting conditions. The biocathode was prepared with bilirubin oxidase from *Bacillus pumilus* (*Bp*‐BOD),^32^ a stable multi‐copper oxidase used previously in biofuel cells,^18^ by drop‐casting a *Bp*‐BOD stock solution (borate buffer, 50 mm, pH, pH 9, 54.75 mg mL^−1^) onto a carbon cloth‐based gas‐diffusion layer equipped with a conducting microporous Nafion/Teflon/carbon layer with enhanced surface area (for a detailed description of the immobilization process, see the Experimental Section). In cyclic voltammograms, maximum absolute currents for O_2_ reduction of approximately 180 μA were observed when the gas‐diffusion electrode was exposed to air (Figure S5 in the Supporting Information). These values are significantly higher than those obtained for the polymer/hydrogenase‐modified glassy carbon electrodes exhibiting maximum absolute currents <80 μA for all hydrogenases.

Membrane‐free biofuel cells prepared with the gas‐diffusion *Bp*‐BOD‐based biocathode in combination with P(N_3_MA‐BA‐MA)‐vio/*wt*‐[NiFeSe] (Figure S6 a in the Supporting Information), P(N_3_MA‐BA‐MA)‐vio/G491A (b), and P(N_3_MA‐BA‐MA)‐vio/G491S (c) bioanodes showed OCV values of 1.06, 1.05, and 1.06 V, respectively. As expected, all biofuel cell assemblies show similar or even identical OCV values (note that all electrodes show very similar enzyme and polymer loadings), indicating again the benefits of the two variants compared with typically used O_2_‐tolerant hydrogenases with higher redox potentials. The maximum power density with respect to the electrode surface area of the bioanode was observed at 0.8 V for all fuel cells and was estimated to be 340 μW cm^−2^ (*wt*‐[NiFeSe]), 325 μW cm^−2^ (G491A), and 271 μW cm^−2^ (G491S). The values are similar to other polymer‐based biofuel cells using [NiFe]^8^ and [FeFe]^9^ hydrogenases.

Recently, we showed that the use of gas‐diffusion layers modified with polymer/*wt*‐[NiFeSe] and polymer/[NiFe] films displayed enhanced power output owing to an enhanced mass transport of the gaseous substrate H_2_ towards the bioanode.^12^ Current densities for the bioanode of close to 8 mA cm^−2^ and power densities of 3.8 mW cm^−2^ for biofuel cells with a bilirubin oxidase‐modified gas‐diffusion biocathode were observed.^12^ To demonstrate the relevance of the O_2_‐tolerant [NiFeSe] variants, carbon cloth‐based gas‐diffusion layers were first modified with P(GMA‐BA‐PEGMA)‐vio [poly(glycidyl methacrylate‐*co*‐butyl acrylate‐*co*‐poly(ethylene glycol)methacrylate)‐vio; for the structure and synthesis of this polymer, see Figure S1 in the Supporting Information and Ref. 12, respectively] films followed by the immobilization of an active P(N_3_MA‐BA‐GMA)‐vio/G491S layer [Figure 1 b; for a detailed description of the preparation process see the Experimental Section; electrodes are denoted as P(GMA‐BA‐PEGMA)‐vio//P(N_3_MA‐BA‐GMA)‐vio/G491S; owing to the limited amount of enzyme, only the variant G491S was used for the preparation of a H_2_‐oxidation gas‐diffusion layer].

The less hydrophilic viologen‐modified polymer P(GMA‐BA‐PEGMA)‐vio acts as an adhesion layer between the hydrophilic active layer and the hydrophobic carbon cloth surface.^12^ Moreover, the underlying redox polymer layer prevents contribution from DET between the enzyme and the porous electrode surface and excludes high‐potential inactivation.^12^

**Figure 5:**
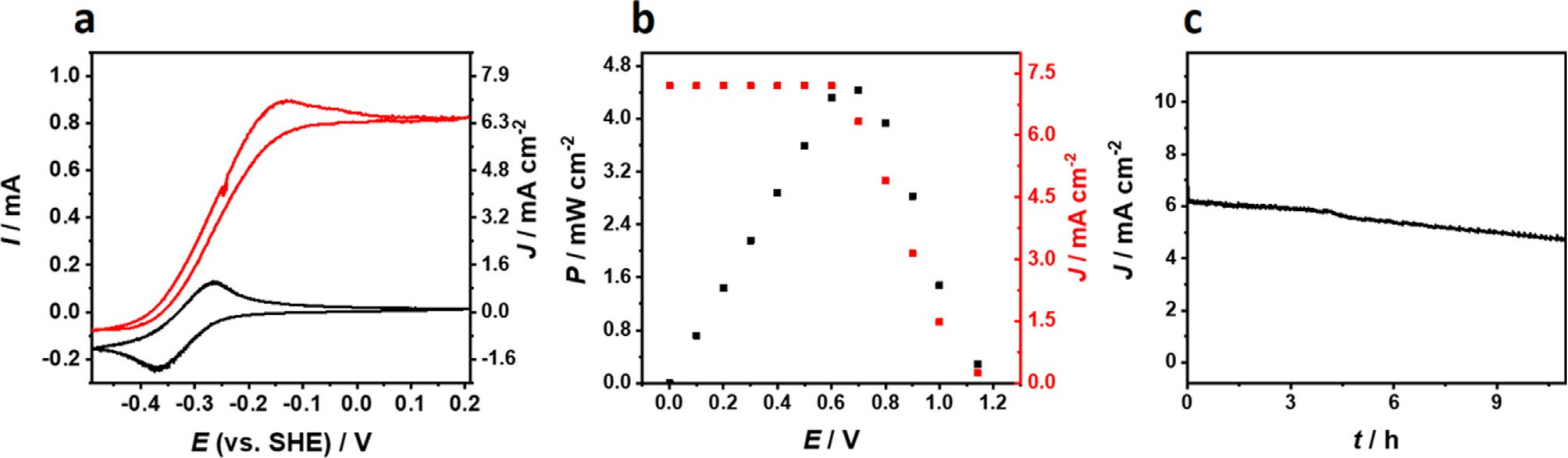
Characterization of (a) the P(GMA‐BA‐PEGMA)‐vio//P(N_3_MA‐BA‐GMA)‐vio/G491S‐based gas‐diffusion bioanode and (b, c) biofuel cells equipped with a *Mv*‐BOD‐based gas‐diffusion O_2_‐reducing biocathode (operated in 100 % O_2_) in 0.1 m phosphate buffer, pH 7.4. (a) Cyclic voltammograms of the gas‐diffusion P(N_3_MA‐BA‐GMA)‐vio/G491S bioanode in the absence (black curve) and presence of H_2_ (red curve); scan rate=5 mV s^−1^. (b) Current density (red squares, right ordinate) and power density (black squares, left ordinate, with respect to the geometric surface area of the modified part of the bioanode, ≈0.126 cm^−2^). (c) Operational stability of the biofuel cell over 10 h at 0.7 V. Nominal biocatalyst loading: 8.4 nmol cm^−2^/1.06 nmol electrode^−1^.

Under gas‐diffusion conditions, the bioanode showed absolute H_2_‐oxidation currents of approximately 0.8 mA (Figure 5 a). The modified surface area of the carbon cloth‐based bioanode has a diameter of approximately 4 mm, which results in a surface area of the active layer of approximately 0.126 cm^−2^, and thus maximum current densities of 6.3 mA cm^−2^ were achieved. The values are similar to previously reported polymer‐based gas‐diffusion systems equipped with *wt*‐[NiFeSe] and [NiFe] hydrogenases (Table S1 in the Supporting Information). However, care must be taken when comparing current densities measured with porous electrodes. Because of the 3D structure of the electrodes, the real surface is often unknown. Hence, the catalyst loading is a better value for comparison. For the G491S‐based electrodes, the catalyst loading is 8.4 nmol cm^−2^/1.06 nmol electrode^−1^. Interestingly, for the *wt*‐[NiFeSe] hydrogenase, current densities of only 5.3 mA cm^−2^ were observed with a substantially higher catalyst loading of 27.0 nmol cm^−2^/3.4 nmol electrode^−1^ as reported in our previous work (see Ref. 12 and Table S1 in the Supporting Information), which largely exceeds the values of the G491S variant at almost identical overall polymer loading [*wt*‐[NiFeSe]: 230 μg electrode^−1^ (previous work, Ref. 12); G491S: 260 μg electrode^−1^]. At a lower catalyst loading of 12.1 nmol cm^−2^/1.53 nmol electrode^−1^ (polymer loading 230 μg electrode^−1^), which is only slightly higher than the loading of the G491S enzyme, the *wt*‐[NiFeSe] shows a *J*_max_ value of only 3.6 mA cm^−2^ (see Ref. 12). This effect might be related to an improved incorporation of the G941S variant in the polymer film when immobilized on the rather hydrophobic carbon cloth‐based electrodes. A stronger interaction prevents leaching of the enzyme and thus ensures a higher local concentration of the biocatalyst during the experiment. In addition, a loss of activity in the immobilized state for the wild type may also contribute to a reduced electrode activity. An effect of different polymer‐to‐enzyme ratios can be ruled out because almost identical polymer loadings were used for all experiments. However, the effect seems to be specific for the porous, hydrophobic carbon cloth electrodes because the wild type shows a higher activity on flat glassy carbon electrodes (see Figure 2 and Figure S2 in the Supporting Information).

To evaluate the performance of the gas‐diffusion P(GMA‐BA‐PEGMA)‐vio//P(N_3_MA‐BA‐GMA)‐vio/G491S electrode in an all‐gas‐diffusion membrane‐free H_2_/O_2_ biofuel cell, the bioanode was combined with an O_2_‐reducing biocathode modified with bilirubin oxidase from *Myrothecium verrucaria* (*Mv*‐BOD, for comparison purposes because it was used in our previously reported experiments^12^). For the immobilization of *Mv*‐BOD, the carbon cloth was first modified with 2‐ABA (2‐amino benzoic acid) to ensure a proper orientation of the enzyme on the electrode surface. The modifier was anchored in an electrochemical grafting process by applying an oxidative potential pulse.^33^ The *Mv*‐BOD was then immobilized by means of a conventional drop‐casting process and was operated in the DET regime.^12^, ^33^ A high catalyst loading was used to ensure anode‐limiting conditions (nominal enzyme loading: 1.2 mg electrode^−1^). Absolute currents under gas‐diffusion conditions (100 % O_2_) reached approximately 2 mA (Figure S7 in the Supporting Information), which largely outperforms the bioanode (≈0.8 mA, Figure 5 a). The fully assembled H_2_/O_2_ biofuel cell (Figure 5 b) showed an OCV of 1.14 V, which is slightly higher than the values obtained on glassy carbon electrodes (1.05–1.06 V); this might be attributed to the slightly lower overpotential for O_2_ reduction of *Mv*‐BOD compared with *Bp*‐BOD.^34^ The maximum power density was reached at 0.7 V and was estimated to be 4.4 mW cm^−2^. This value even outperforms our previously reported value for the [NiFe]‐based biofuel cell (3.6 mW cm^−2^)^12^ and—to the best of our knowledge—sets a new benchmark for a biofuel cell using redox‐polymer‐based bioanodes (Table S1 in the Supporting Information). Moreover, the catalyst loading is significantly lower than the [NiFe] system (31.8 nmol cm^−2^/4 nmol electrode^−1^)^12^ reported previously (Table S1 in the Supporting Information).

Cyclic voltammograms (Figure S8 in the Supporting Information) measured before and after biofuel cell operation showed similar values for the bioanode, with the slightly higher currents after the biofuel cell test most likely as a result of changed diffusion properties inside the polymer/enzyme layer, for example, owing to swelling and/or slightly changed local pH values, which will affect the overall activity of the enzyme. In contrast, the current of the biocathode was slightly decreased after the biofuel evaluation (Figure S7 in the Supporting Information). This again highlights the high stability of the bioanode in a membrane‐free biofuel cell under anode‐limiting conditions. The operational stability of the biofuel cell was tested at a constant load of 0.7 V (Figure 5 c). After 10 h of continuous operation, 75 % of the initial current density remained. Cyclic voltammograms measured after the long‐term experiment showed significantly lower currents for the bioanode (Figure S8 in the Supporting Information) and the biocathode (Figure S7 in the Supporting Information). We want to emphasize that the amplitudes of the polymer signals (Figure S7 in the Supporting Information, dashed black curve) were also decreased compared with the voltammograms measured with the freshly prepared electrode. Thus, not only does deactivation/decomposition of the enzyme contribute to the decreased activity after long‐term operation, but the loss of immobilization matrix may also have an effect. Nevertheless, the bioanode shows an outstanding performance and demonstrates the potential applicability of G941S (enhanced O_2_ tolerance) as a highly active and stable catalyst in a membrane‐free biofuel cell device. Moreover, the proposed H_2_‐oxidation bioanodes combine the advantages of the protection matrix (O_2_ quenching; no high‐potential deactivation) and the enhanced enzyme stability of the hydrogenase variants (blocking of O_2_ access) in accordance with the mechanism depicted in Figure 1 b and thus demonstrate a triple‐protection system for the high‐current‐density H_2_‐oxidation bioanodes.

## Conclusions

The two [NiFeSe] variants show a higher O_2_ tolerance than the wild type in the immobilized state, which is in line with results reported for the enzymes operated in a direct electron transfer regime.^27^ In combination with the redox polymer‐based protection matrix, the proposed bioanodes reveal a triple‐protection system that ensures stable operation. Moreover, we could demonstrate that the two [NiFeSe] variants show similar performance as the wild type and as [NiFe] as well as [FeFe] hydrogenases when incorporated into a conventional redox‐polymer‐based biofuel cell. In addition, the use of gas‐diffusion layers ensured high substrate transport towards the active polymer/enzyme layer, allowing H_2_‐oxidation currents of approximately 6.3 mA cm^−2^ for the G491S variant at comparatively low catalyst loadings. Combination of the gas‐diffusion bioanode with a gas‐diffusion O_2_‐reducing biocathode allowed for the fabrication of a H_2_/O_2_‐powered biofuel cell with benchmark performance in a membrane‐free configuration. We conclude that the novel O_2_‐tolerant [NiFeSe] variants are promising candidates for biofuel cell applications, demonstrating that enzyme engineering is indeed a powerful tool, which may be used to not only overcome sensitivity issues but also to further enhance the activity and stability of biocatalysts.

## Experimental Section

### Chemicals and materials

All chemicals and materials were purchased from Sigma–Aldrich, Alfa‐Aesar, VWR, Acros‐Organics, or Fisher Scientific and were used as received (reagent or analytical grade) except where otherwise noted. For the preparation of all aqueous solutions, deionized water from a Millipore water‐purification system was used. The synthesis and characterization of the redox polymer poly(3‐azido‐propyl methacrylate‐*co*‐butyl acrylate‐*co*‐glycidyl methacrylate)‐vio [P(N_3_MA‐BA‐GMA)‐vio, with vio=(1‐(5‐hexyn‐1‐yl)‐1′‐methyl‐4,4′‐bipyridinium] was described previously in Ref. 10. It was used as an aqueous solution with a concentration of 7.3 mg mL^−1^. The synthesis and characterization of the less hydrophilic redox polymer poly(glycidyl methacrylate‐*co*‐butyl acrylate‐*co*‐poly(ethylene glycol) methacrylate)‐vio [P(GMA‐BA‐PEGMA)‐vio] was described previously in Ref. 12. It was used as an aqueous solution with a concentration of 7.5 mg mL^−1^.

### Enzymes

The isolation and purification of the wild‐type [NiFeSe] hydrogenase from *D. vulgaris* Hildenborough (*wt*‐[NiFeSe]) was described previously in Ref. 28. The activity for H_2_ uptake was measured as (4850±260) s^−1^.^27^ The enzyme was stored in Tris‐HCl buffer, 20 mm, pH 7.6 at −80 °C at a concentration of 170 μm. The preparation of the [NiFeSe] variants G491A and G491S is described in Ref. 27. Their H_2_ uptake was measured to be (1918±119) s^−1^ (G491A) and (2416±387) s^−1^ (G491S). The [NiFeSe] variants were stored in Tris‐HCl, 20 mm, pH 7.6 at −80 °C (G491A: 82.96 μm; G491S: 53 μm).

Bilirubin oxidase from *Bacillus pumilus* (*Bp*‐BOD) was isolated and purified according to protocols reported in Ref. 32. The protein was stored in 50 mm borate buffer, pH 9, at −80 °C; concentration=54.75 mg mL^−1^; activity=713 U mg^−1^. Bilirubin oxidase from *Myrothecium verrucaria* (*Mv*‐BOD, lyophilized powder, 15–65 U mg^−1^ protein) was obtained from Sigma–Aldrich and stored at −20 °C as a powder. For electrode modification, the enzyme was dissolved in phosphate buffer, 0.1 m, pH 7.3, at a concentration of 15 mg mL^−1^.

### Electrochemical experiments

All electrochemical experiments were conducted under the corresponding atmosphere (argon, hydrogen, oxygen, and their mixtures) and at room temperature by using a Gamry Reference 600 potentiostat in a three‐electrode configuration with an Ag/AgCl/3 m KCl reference electrode. All potentials were rescaled to the standard hydrogen electrode (SHE) according to the equation *E*_SHE_=*E*_Ag/AgCl/3 m KCl_+210 mV. Phosphate buffer (0.1 m, pH 7.3) was used as electrolyte for all experiments. For cyclic voltammetric and chronoamperometric experiments, a Pt counter electrode and modified glassy carbon disk working electrodes (3 mm) were used. The latter were polished by using, first, diamond particles (3 μm) followed by Al_2_O_3_ powder (1 μm, then 0.3 μm) following standard protocols. Measurements under gas‐diffusion conditions were performed in a homemade glass cell^12^ with carbon cloth‐based gas‐diffusion electrodes [MTI, carbon foam sheet, porous C, 0.454 mm thick, ≈10 mL cm^−2^ s^−1^, porosity ≈31 μm coated on one side with a conductive Nafion/Teflon‐based microporous film (50 μm), carbon content 5 mg cm^−2^, EQ‐bcgdl‐1400S‐LD]. Thermal mass flow controllers (GFC17, Aalborg Instruments and Controls) were used to adjust the desired atmosphere and gas mixtures with predefined compositions (for compositions of the gas feed, see the main text and figures). The back of the gas‐diffusion electrode was exposed to the corresponding gas atmosphere (bioanode) or to air/O_2_ (biocathode). During the experiments in gas‐breathing mode, the electrochemical cell/electrolyte was continuously purged with an argon stream to prevent permeation of O_2_ into the bulk electrolyte. For characterization of the biofuel cells, power curves were measured by stepped potential chronoamperometric experiments to minimize contributions from capacitive charging currents. After each potential step, steady‐state currents were used to calculate the corresponding power values.

#### Modification of glassy carbon electrodes with hydrogenase/polymer films

All films were prepared by means of a standard drop‐casting process. For this, stock solutions of the polymer P(N_3_MA‐BA‐GMA)‐vio and the corresponding hydrogenase variant were prepared: 4 μL of an aqueous P(N_3_MA‐BA‐GMA)‐vio solution (7.3 mg mL^−1^) were mixed with 3 μL of phosphate buffer (0.1 m, pH 7.3), and the corresponding hydrogenase was added (*wt*‐[NiFeSe]: 0.5 μL, 170 μm in Tris‐HCl buffer, 20 mm, pH 7.6; G491A: 1 μL, 82.96 μm in Tris‐HCl buffer; G491S: 1.56 μL, 53 μm in Tris‐HCl buffer). For electrode modification, 1.3 μL of the stock solution was drop‐cast onto the 3 mm glassy carbon disk electrode. The modified electrodes were incubated overnight at 4 °C and air dried for 1 h prior to use. In a typical experiment, three electrodes were modified from the same stock solution.

#### Modification of carbon cloth electrodes with hydrogenase/polymer films

First, 20 μL of the polymer P(GMA‐BA‐PEGMA)‐vio (7.5 mg mL^−1^ in water) was drop‐cast on the microporous side of the carbon cloth electrode and dried overnight at room temperature. Subsequently, 20 μL of G491S (53 μm in Tris‐HCl, 20 mm, pH 7.6) was mixed with 15 μL of P(N_3_MA‐BA‐GMA)‐vio (7.3 mg mL^−1^ in water) and drop‐cast onto the already existing polymer spot (diameter of ≈4 mm). The electrode was dried overnight at 4 °C.

#### Modification of carbon cloth electrodes with *Bp*‐BOD

The bare carbon cloth was pre‐wetted with ethanol on both sides and rinsed with water. Then, the microporous side of the gas‐diffusion layer was modified with 20 μL of the *Bp*‐BOx solution (54.75 mg mL^−1^ in borate buffer, 50 mm, pH 9). The electrode was dried overnight at 4 °C.

#### Modification of carbon cloth electrodes with *Mv*‐BOD

For the preparation of the *Mv*‐BOD‐based cathode, the microporous side of the ethanol‐treated carbon cloth gas‐diffusion electrode was first modified with 2‐amino benzoic acid (2‐ABA) in an electrochemical grafting process in 0.1 m KCl/5 mm 2‐ABA/water by applying a potential pulse of +0.8 V vs. Ag/AgCl/3 m KCl for 60 s according to procedures reported in Refs. 12, 33. The modified electrode was rinsed with water and further modified with 120 μL of an aqueous *Mv*‐BOD solution (10 mg mL^−1^, nominal enzyme loading: 1.2 mg electrode^−1^) and dried at 4 °C overnight.

## Conflict of interest

*The authors declare no conflict of interest*.

## Supporting information

As a service to our authors and readers, this journal provides supporting information supplied by the authors. Such materials are peer reviewed and may be re‐organized for online delivery, but are not copy‐edited or typeset. Technical support issues arising from supporting information (other than missing files) should be addressed to the authors.

Supplementary
