# Supplementary material for: Redox‐Polymer‐Wired [NiFeSe] Hydrogenase Variants with Enhanced O2 Stability for Triple‐Protected High‐Current‐Density H2‐Oxidation Bioanodes
Source: ChemSusChem. 2020 Jun 8;13(14):3627–35. doi: 10.1002/cssc.202000999 (PMC7497094; doi:10.1002/cssc.202000999)
Supplement: Supplementary file 1 — Supplementary [file CSSC-13-3627-s001.pdf]

ChemSusChem

Supporting Information

Redox-Polymer-Wired [NiFeSe] Hydrogenase Variants with Enhanced O_2 Stability for Triple-Protected High-Current-Density H_2-Oxidation Bioanodes

Adrian Ruff,*[a] Julian Szczesny,[a] Maria Vega,[b] Sonia Zacarias,[c] Pedro M. Matias,[c, d] Sébastien Gounel,[e] Nicolas Mano,[e] Inês A. C. Pereira,[c] and Wolfgang Schuhmann*[a]

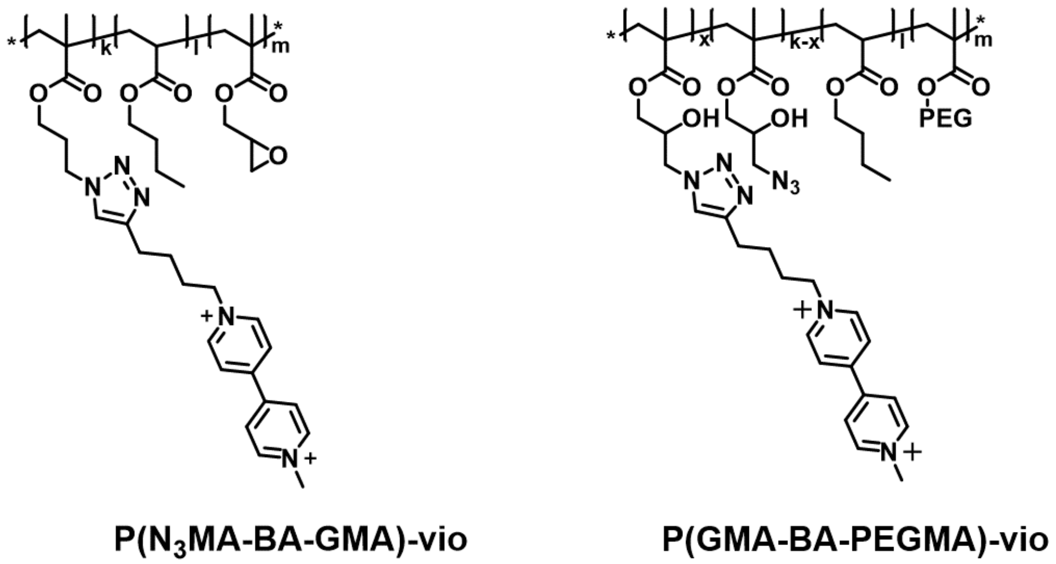

Figure S1: Molecular structures of the redox polymers P(N_3MA-BA-GMA)-vio (left) and P(GMA-BA-PEGMA)-vio (right). The actual compositions of the polymers are k = 71 mol %, l = 20 and 9 mol % (P(N_3MA-BA-GMA)-vio) and k = 65 mol%, l = 32 mol%, m = 3 mol%, x ≈ k, only residual k-units in the final polymer (P(GMA-BA-PEGMA)-vio). For synthesis and characterization of the polymers P(N_3MA-BA-GMA)-vio and P(GMA-BA-PEGMA)-vio the reader is referred to refs. [1] and [2], respectively.

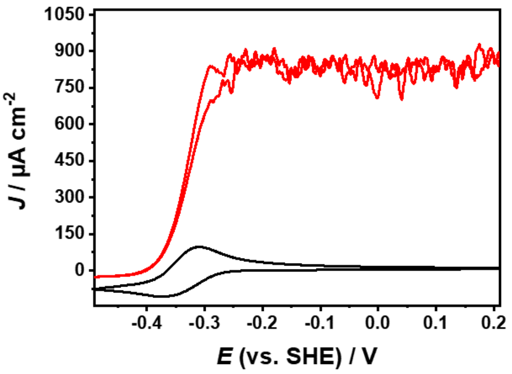

Figure S2: Cyclic voltammogram of a P(N_3MA-BA-GMA)-vio/*wt*-[NiFeSe] modified glassy carbon electrode in phosphate buffer (0.1 M, pH 7.4) at room temperature in the absence of H_2 (100 % argon, black line) and presence of H_2 (100 % H_2, red curve). Scan rate = 10 mV s^{-1}. Gases were purged through the electrolyte. The fluctuations at high potentials (< -0.3 V vs. SHE) are attributed to local fluctuations in substrate transport and/or local pH changes during turnover.[1]

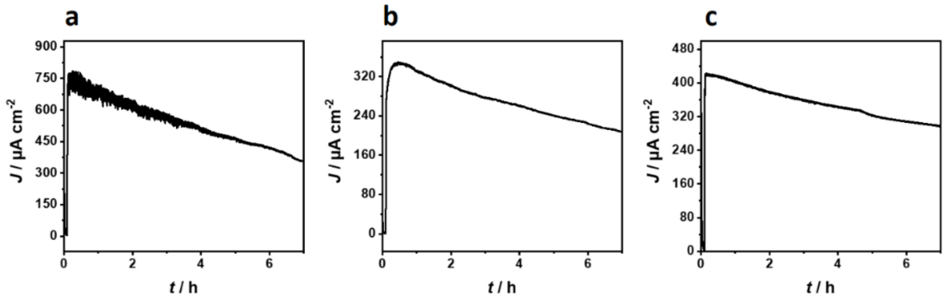

Figure S3: Chronoamperometric experiments with P(N_3MA-BA-GMA)-vio/*wt*-[NiFeSe] (**a**), P(N_3MA-BA-GMA)-vioG491A (**b**) and P(N_3MA-BA-GMA)-vio/G491S (**c**) modified glassy carbon electrodes under continuous turnover (100 % H_2, purged through electrolyte). Working conditions: E_{appl} = +160 mV vs. SHE; phosphate buffer, 0.1 M, pH 7.4; room temperature.

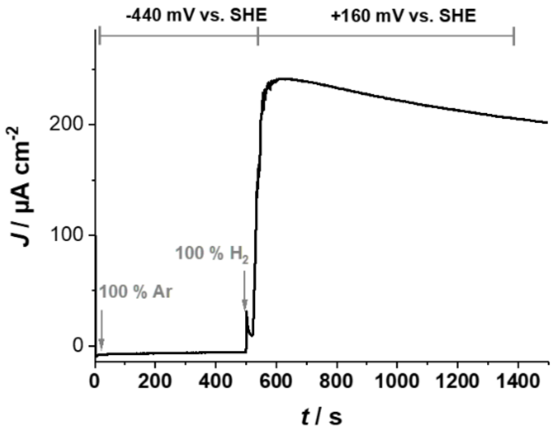

Figure S4: Chronoamperometric experiment with a anaerobically deactivated P(N_3MA-BA-GMA)-vio/*wt*-[NiFeSe] film immobilized at a glassy carbon disk electrode (3 mm Ø). First, a potential of -440 mV vs. SHE was applied for 500 s to fully reduce the viologen modified polymer (enzyme is reactivated via reduction by the polymer). After switching the potential to +160 mV vs. SHE (t > 500 s) H_2 oxidation currents indicate successful reactivation. Working conditions: phosphate buffer, 0.1 M, pH 7.3, room temperature, electrodes were deactivated by extensive exposure to O_2 until a H_2 oxidation current was absent.

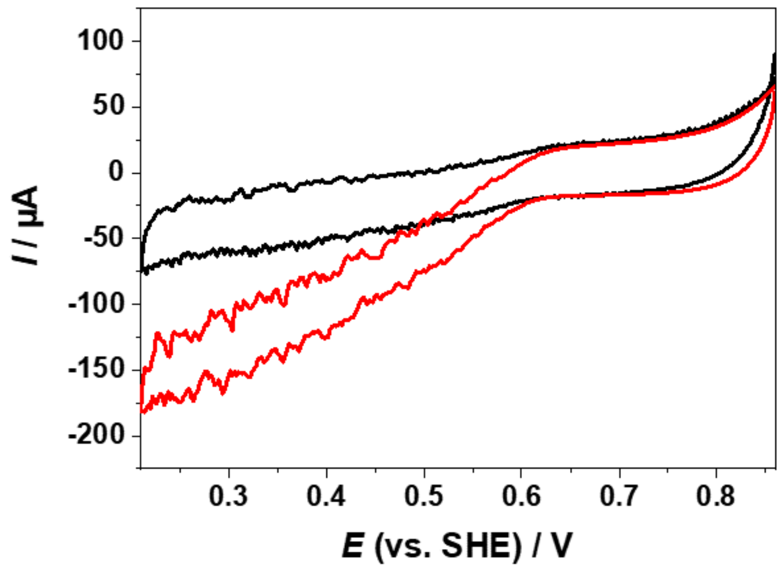

Figure S5: Cyclic voltammograms of a *Bp*-BOD modified carbon cloth (microporous side) gas diffusion layer in 0.1 M phosphate buffer (pH 7.3) under argon (purged through electrolyte, black curve) and under gas diffusion conditions in air (red curve). Conditions: scan rate = 5 mV s^{-1}; room temperature.

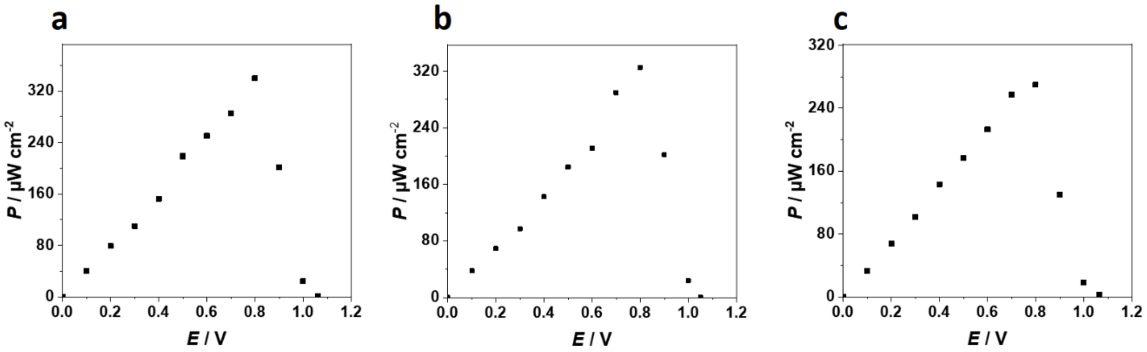

Figure S6: Characterization of the P(N_3MA-BA-GMA)-vio/wt-[NiFeSe] (a), P(N_3MA-BA-GMA)-vioG491A (b) and P(N_3MA-BA-GMA)-vio/G491S (c) based H_2/O_2 biofuel cells. The modified glassy carbon bioanodes were combined with a *Bp*-BOD modified carbon cloth gas diffusion layer to ensure anode limiting conditions. Working conditions: phosphate buffer, 0.1 M, pH 7.3. Ar/H_2 was purged through the cell. The biocathode was operated under gas diffusion conditions in air; measurements were performed at room temperature.

Table S1: Current densities of polymer/hydrogenase gas diffusion bioanodes and their power densities when incorporated into H_2/O_2 biofuel cells. For comparison purposes, some benchmark values for devices operate in DET mode are given.

Hydrogenase (polymer)	MET or DET regime	catalyst loading	J_{max}/mA cm^{-2} (electrode substrate)	P_{max}/mW cm^{-2} at r.t. or 25 °C (biocathode)	Ref.
***wt*-[NiFeSe]** (P(GMA-BA-PEGMA)-vio and P(N3MA-BA-GMA)-vio) $^{+}$	MET	27.0 nmol cm^{-2}/3.4 nmol electrode^{-1}	5.3 mA cm^{-2} (carbon cloth)	-	2
***wt*-[NiFeSe]** (P(GMA-BA-PEGMA)-vio and P(N3MA-BA-GMA)-vio) $^{+}$	MET	12.1 nmol cm^{-2}/1.53 nmol electrode^{-1}	3.6 mA cm^{-2} (carbon cloth)	1.9 mW cm^{-2} at 0.7 V (*Mv*-BOD on carbon cloth)	2
***Dv*MF-[NiFe]** (P(GMA-BA-PEGMA)-vio and P(N3MA-BA-GMA)-vio) $^{+}$	MET	31.8 nmol cm^{-2}/ 4 nmol electrode^{-1}	7.9 mA cm^{-2} (carbon cloth)	3.6 mW cm^{-2} (*Mv*-BOD on carbon cloth)	2
**G491S (P(GMA-BA-PEGMA)-vio and P(N3MA-BA-GMA)-vio)** $^{+}$	**MET**	**8.4 nmol cm^{-2}/1.06 nmol electrode^{-1}**	**6.3 mA cm^{-2}** (carbon cloth)	**4.4 mW cm^{-2} at 0.7 V (Mv-BOD on carbon cloth)**	**This work**
**[NiFeSe]** from *Desulfomicrobium baculatum*	DET	14 pmol cm^{-2}	1.9 mA cm^{-2} (functionalized MWCNTs)	0.89 mW cm^{-2} at 0.80 V (*Mv*-BOD on MWCNTs)	3
***Dv*MF-[NiFe]**	DET	53 nmol cm^{-2}/ 5 nnmol electrode^{-1}	15.8 mA cm^{-2} (Ketjen black modified waterproof carbon paper)	6.1 mW cm^{-2} at 0.72 V (*Mv*-BOD on Ketjen black modified waterproof carbon paper)	4
**[NiFe]** from *Hydro-genovibrio marinus* and *Dv*MF-[NiFe]	DET	1.1 mg cm^{-2}/ 1.5 mg electrode^{-1}	≈20 mA cm^{-2} (Ketjen black modified waterproof carbon paper	8.4 mW cm^{-2} at 0.7 V (*Mv*-BOD on Ketjen black modified waterproof carbon paper)$^{++}$	5

$^{+}$ polymer double layer system was employed to avoid DET; $^{++}$ theoretical value calculated from the individual half cells.

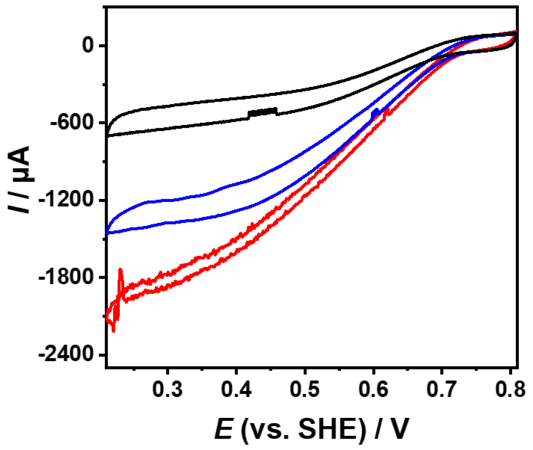

Figure S7: Cyclic voltammograms of a *Mv*-BOD modified carbon cloth (microporous side, 2-ABA modified) gas diffusion biocathode measured in phosphate buffer (0.1 M, pH 7.4) under gas diffusion conditions (100 % O_2 gas feed) before biofuel cell measurements (red curve), after biofuel cell measurement (blue curve) and after testing of the operational stability of the membrane-free gas diffusion biofuel cell (black curve). Conditions: scan rate = 5 mV s^{-1}, room temperature.

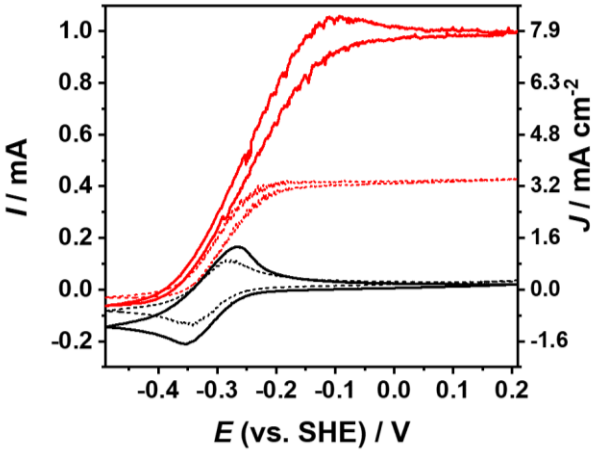

Figure S8: Cyclic voltammetric characterization of the P(N_3MA-BA-GMA)-vio/G491S carbon cloth based bioanode after the biofuel cell test under gas diffusion conditions (Ar: black solid line; H_2: red solid line) and after the operational stability test (Ar: black dashed line; H_2: red dashed line); for values measured with the freshly prepared electrode see Figure 4a in the main text. Working conditions: phosphate buffer, 0.1 M, pH 7.4; scan rate = 5 mV s^{-1}, room temperature.

References

1 A. Ruff, J. Szczesny, S. Zacarias, I. A. C. Pereira, N. Plumeré and W. Schuhmann, *ACS Energy Lett.*, 2017, **2**, 964.

2 J. Szczesny, N. Marković, F. Conzuelo, S. Zacarias, I. A. C. Pereira, W. Lubitz, N. Plumeré, W. Schuhmann and A. Ruff, *Nat. Commun.*, 2018, **9**, 4715.

3 S. Gentil, S. M. Che Mansor, H. Jamet, S. Cosnier, C. Cavazza and A. Le Goff, *ACS Catal.*, 2018, **8**, 3957.

4 H.-q. Xia, K. So, Y. Kitazumi, O. Shirai, K. Nishikawa, Y. Higuchi and K. Kano, *J. Power Sources*, 2016, **335**, 105.

5 K. So, Y. Kitazumi, O. Shirai, K. Nishikawa, Y. Higuchi and K. Kano, *J. Mater. Chem. A*, 2016, **4**, 8742.
